# Effect of Sex, Age, and Cardiovascular Risk Factors on Aortoiliac Segment Geometry

**DOI:** 10.3390/jcm13061705

**Published:** 2024-03-15

**Authors:** Ádám Szőnyi, György Balázs, Balázs Bence Nyárády, Márton Philippovich, Tamás Horváth, Edit Dósa

**Affiliations:** 1 Heart and Vascular Center, Semmelweis University, 1122 Budapest, Hungary; szonyi.adam@stud.semmelweis.hu (Á.S.); balazs.gyorgy.laszlo@semmelweis.hu (G.B.); nyarady.balazs.bence@semmlweis.hu (B.B.N.); philippovich.marton@semmelweis.hu (M.P.); 2 Heim Pál National Pediatric Institute, 1089 Budapest, Hungary; 3 Research Center for Sport Physiology, Hungarian University of Sports Science, 1123 Budapest, Hungary; horvath2.tamas@tf.hu

**Keywords:** aortoiliac arteries, arterial geometry, tortuosity index, absolute average curvature, sex, age, cardiovascular risk factors

## Abstract

**Background:** To investigate the geometry of the aortoiliac (AI) segment and its correlation with sex, age, and cardiovascular (CV) risk factors. **Methods:** Abdominal and pelvic CTA/MRA scans of 204 subjects (120 males; median age: 53 [IQR, 27–75] years) without AI steno-occlusive disease or scoliosis were retrospectively analyzed. The participants were enrolled consecutively, ensuring the representation of each age decade. An in-house written software was developed to assess AI elongation using the tortuosity index (TI) and absolute average curvature (AAC). Aortic bifurcation angle, common iliac artery (CIA) take-off and planarity angles, bifurcation asymmetry, and deviation from optimal bifurcation were calculated and evaluated. Demographic data, CV risk factors, and medical history were collected from electronic health records. **Results:** The elongation of the iliac arteries was more pronounced in males (TI: left CIA, *p* = 0.011; left EIA, *p* < 0.001; right CIA, *p* = 0.023; right EIA, *p* < 0.001; AAC: left EIA, *p* < 0.001; right EIA, *p* = 0.001). Age significantly influenced TI and AAC in all AI segments (all *p* < 0.001), but was also positively associated with the aortic bifurcation angle (*p* < 0.001), both CIA planarities (left, *p* < 0.001; right, *p* = 0.002), aortic bifurcation asymmetry (*p* = 0.001), and radius discrepancy (*p* < 0.001). Significant positive correlations were found between infrarenal aortic TI/AAC and chronic kidney disease (CKD) (*p* = 0.027 and *p* = 0.016), AAC of both CIAs and hypertension (left, *p* = 0.027; right, *p* = 0.012), right CIA take-off angle and CKD (*p* = 0.031), and left CIA planarity and hyperlipidemia (*p* = 0.006). **Conclusion:** Sex, age, and CV risk factors have a significant effect on the geometry of the AI segment.

## 1. Introduction

Recent epidemiological studies estimate that approximately 230 million people worldwide suffer from peripheral arterial disease (PAD) [1]. As life expectancy continues to increase, PAD is becoming a growing problem, with its prevalence reaching 20% among people over 80 years of age [2]. Lower extremity PAD can cause significant pain, impair mobility, and act as a predictor of potential cardiovascular (CV) events [3]. The aortoiliac (AI) segment is a common site for both steno-occlusive and dilatative forms of PAD, making it a key target for endovascular interventions and open surgical reconstructions. The outcome of invasive treatments is influenced by a number of factors, including classic atherosclerotic risk factors, lesion characteristics, procedural parameters, and specifics of adjunctive conservative therapy. The diameter and course of the aortoiliofemoral axis, as well as the pathological lesions localized there, play a crucial role in the successful delivery of large-lumen devices into the aorta, which are often used during interventions such as transcatheter aortic valve implantation and endovascular repair of aortic aneurysms [4]. It is also known that the elongation of the AI segment is positively related to the complication rate of endovascular repair of aortic aneurysms [5]; however, little information is available on how age and other well-known CV risk factors affect the geometric properties of the AI segment. Our aim was to identify the features of the geometry of the AI segment and the factors that determine them so that the specificities can form the basis of our future anatomical–pathological correlation studies.

## 2. Patients and Methods

### 2.1. Selection of Participants

Our retrospective, two-center study (Heart and Vascular Center, Semmelweis University and Heim Pál National Pediatric Institute) was based on 204 individuals who underwent abdominal and pelvic computed tomography angiography (CTA; Philips Brilliance iCT 256, Philips Medical Systems International B.V., Best, The Netherlands) or magnetic resonance angiography (MRA; Philips Ingenia 1.5 T). The participants were selected consecutively from 2014, ensuring the representation of each age decade (0–10 years, 11–20 years, 21–30 years, 31–40 years, 41–50 years, 51–60 years, 61–70 years, 71–80 years, 81–90 years, and 91–100 years). At least 20 subjects per age group were collected. Candidates with aortoiliac disease or scoliosis were excluded from the study. Demographic data (weight and height), CV risk factors, and medical history were collected from electronic health records. Cardiovascular risk factors included sex, age, obesity, smoking, hypertension, diabetes mellitus, hyperlipidemia, and chronic kidney disease (CKD). The definition of CV risk factors can be found in a publication by Dósa et al. [6]. This study was approved by the Semmelweis University Regional and Institutional Committee of Science and Research Ethics (approval number: 4/2024). All data were fully anonymized prior to access and were exempted from the need for informed consent by the above-mentioned ethics committee.

### 2.2. Image Processing

#### 2.2.1. Arterial Segmentation

Computed tomography angiography and MRA images were segmented to obtain metric data. As a first step, the angiographic images were imported into 3D Slicer (Slicer 3D, 2022, version 5.1.0.20220508. [software]) [7,8,9,10], and semi-automatic segmentation of the abdominal and pelvic arteries was performed using the local thresholding and fast-marching algorithms of the Segment Editor Extra Effects Module [11]. The initial segmentations were smoothed with a 3 mm median filter. The suprarenal aorta, femoral arteries, and all side branches were manually removed, and the resulting segmentations were exported as 3D meshes. The presence of non-aligned faces and vertices necessitated further processing of the preliminary 3D meshes. A custom Python script was created using the Vascular Modeling Toolkit (VMTK; Orobix, Bergamo, Italy; www.vmtk.org [accessed on 10 August 2023]) [12,13] to perform a three-step surface remeshing sequence. First, the surface-connectivity function of VMTK was utilized to eliminate non-manifold edges, separate faces, and vertices. Afterward, a triangular surface remeshing was carried out with a resolution of 0.5 mm. As a last step, 10 iterations of Taubin-smoothing were performed to generate the final topology.

#### 2.2.2. Arterial Geometry

In order to obtain geometric and topological data from the processed 3D surface meshes, the centerline of the vessels had to be generated first. Creation and processing of the centerlines involved several steps including centerline extraction and splitting of the arterial tree according to branching anatomy. The centerline was smoothed with a Laplacian filter (factor of 0.1, 100 iteration steps).

A Python program was developed to ensure reproducibility and to standardize the evaluation. The software executed automatic identification of the infrarenal aorta, common iliac arteries (CIAs), and external iliac arteries (EIAs) and then calculated the geometric characteristics of these vessels. The geometric data were used to quantify the extent of arterial elongation and the branching geometry of the aortic bifurcation.

#### 2.2.3. Arterial Elongation

**Figure 1:**
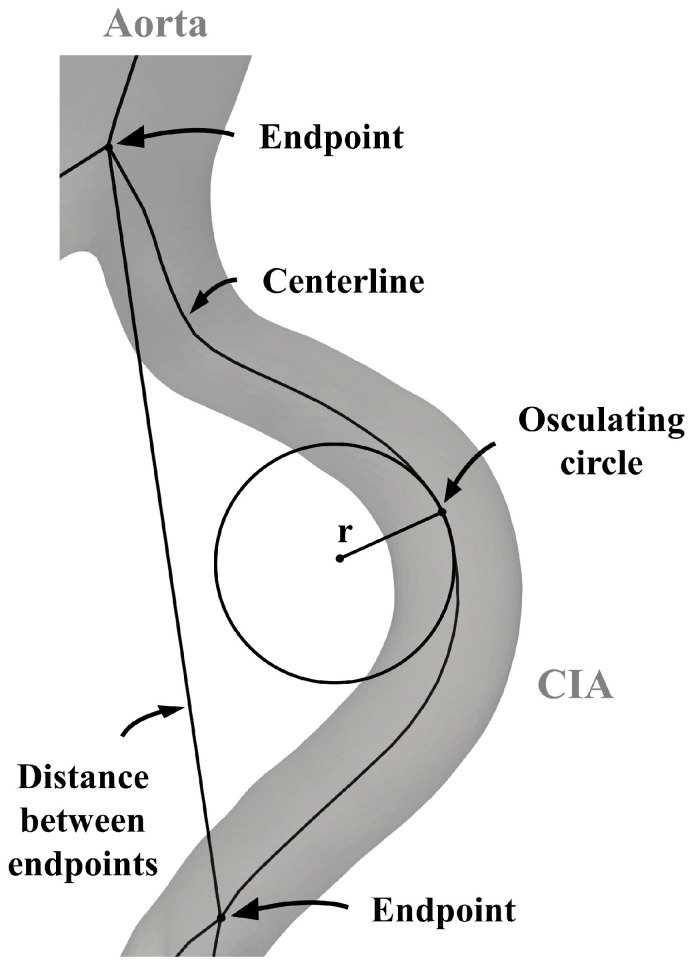
Parameters for the analysis of the aortoiliac segment. CIA—common iliac artery.

As a first step, the length of the arterial segment was determined by calculating the centerline length of each vessel. Arterial elongation was assessed using the tortuosity index (TI) and average absolute curvature (AAC). The TI was determined by comparing the length of the centerline with the shortest distance between its endpoints. Curvature was calculated as the reciprocal of the radius of each osculating circle at 0.5 cm intervals along the centerline. This process yielded multiple curvature values for the arteries, which were then averaged to obtain the ACC for each vessel (Figure 1).

#### 2.2.4. Geometry of Aortic Bifurcation

First, the aortic bifurcation angles were computed; then, cross-sectional areas close to the aortic bifurcation were used to assess whether patient-specific anatomical configurations conformed to the ideal bifurcation topology described by Murray’s law.

**Figure 2:**
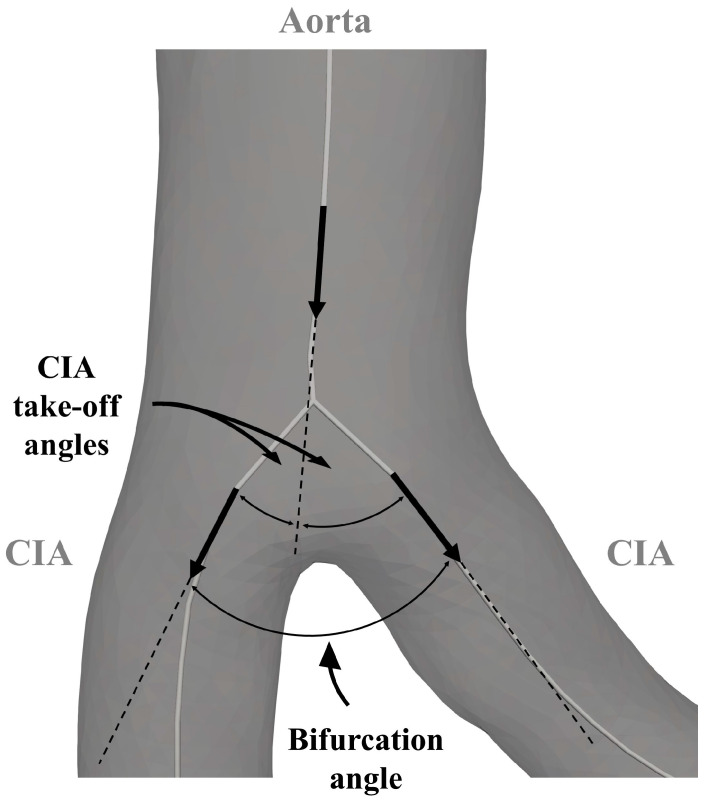
Parameters for the analysis of the aortoiliac segment. CIA—common iliac artery.

**Figure 3:**
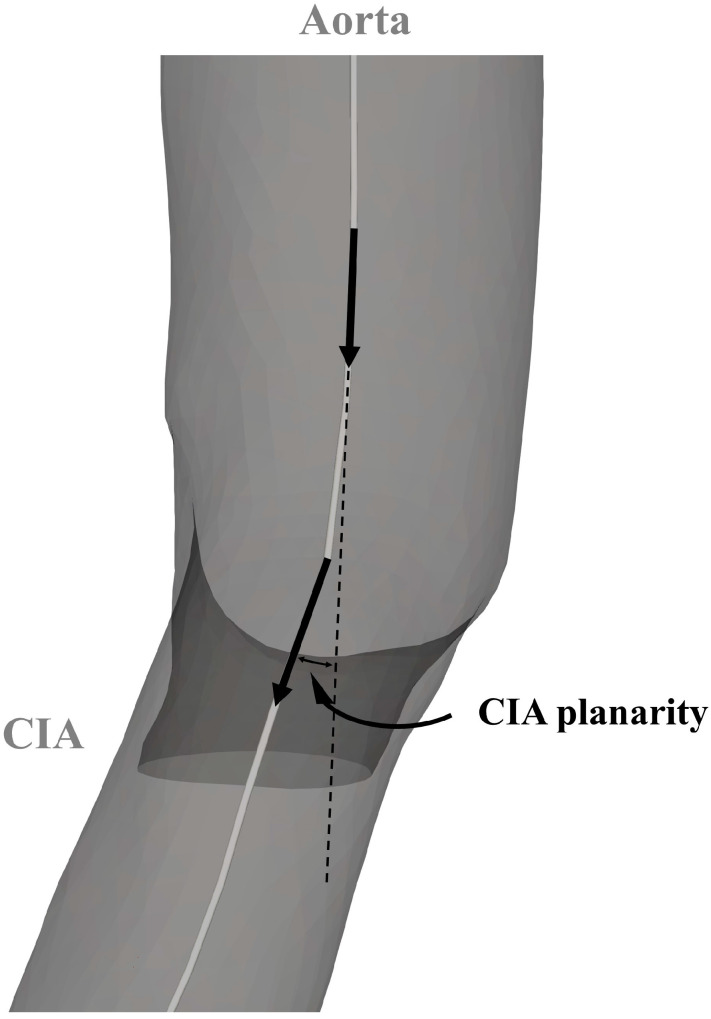
Parameters for the analysis of the aortoiliac segment. CIA—common iliac artery.

A VMTK bifurcation vector system was used to create three vectors representing the two CIAs and the infrarenal aorta. The in-plane orientation of these vectors determined both the bifurcation angle and the take-off angle of the CIA. Common iliac artery planarity was defined by evaluating the out-of-plane orientation relative to the out-of-plane orientation of the infrarenal aorta. The derived angles are illustrated in Figure 2 and Figure 3. The discrepancy between CIA take-off angles was called bifurcation angle asymmetry and was calculated based on an equation proposed by Shakeri et al. [14].

**Figure 4:**
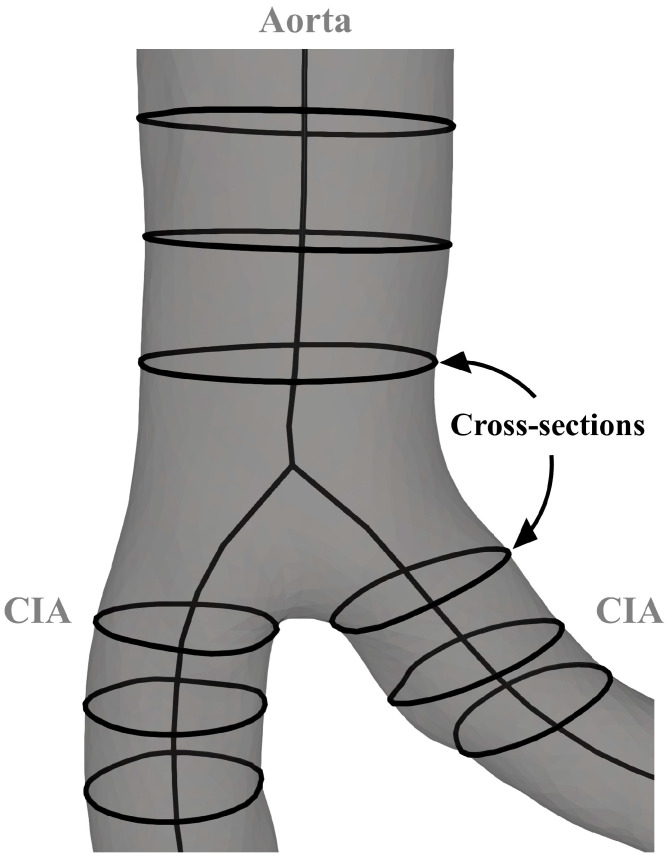
Parameters for the analysis of the aortoiliac segment. CIA—common iliac artery.

The cross-sectional area of each artery was determined at 0.5 cm intervals (Figure 4). Murray’s law states that the sum of the cubes of the radii of the daughter branches is equal to the cube of the radius of the parent artery [15,16]. A multi-step procedure was developed to examine the extent to which the aortic bifurcation in this study group conformed to Murray’s law. First, average cross-sectional areas within 1.5 cm of the aortic bifurcation were recorded. Second, the measured radii (*r_measured_*) of the infrarenal aorta and CIAs were derived from the averaged areas. According to Murray’s law, the ideal radius (*r_ideal_*) of CIAs was calculated from the measured radius of the infrarenal aorta. Finally, to quantify how well the measured radius of the CIAs corresponds to their calculated ideal radius, a radius discrepancy (*r_discrepancy_*) coefficient was implemented.

$$\begin{array}{c} r_{measured}=\sqrt{\frac{A_{measured}}{\pi }}\\ r_{ideal}=\frac{r_{measured}}{\sqrt[{3}]{2}}\\ r_{discrepancy}=\frac{abs\left(r_{CIA_{L}}-r_{ideal}\right)+abs\left(r_{CIA_{R}}-r_{ideal}\right)}{2} \end{array}$$

(*A*—area; *abs*—absolute; *r*—radius; *r_CIA_L_*—measured radius of the left common iliac artery; *r_CIA_R_*—measured radius of the right common iliac artery.)

### 2.3. Statistical Analysis

Descriptive statistics including medians and interquartile ranges (IQRs) were computed for anthropometric data, elongation parameters, and bifurcation metrics. Normality was tested using the Shapiro–Wilk test. The data showed a right-skewed distribution, which made it necessary to normalize them. Normalization was carried out by applying a natural logarithm transformation to all values. The influence of sex was assessed using independent *t*-tests or Mann–Whitney *U* tests, depending on the normality of the variables. To investigate the effect of age on AI morphology, linear regression models were created with arterial elongation and branching geometry as dependent variables. Pearson’s or Spearman’s correlation test was carried out to determine independent correlations between CV risk factors and the parameters of arterial elongation and aortic branching geometry. Statistical significance was determined at the *p* < 0.05 level. Statistical analyses were performed using JASP (JASP Team, Amsterdam, The Netherlands) [17].

## 3. Results

### 3.1. Patient Demographics and Cardiovascular Risk Factors

There were 84 female participants with a median age of 42 years (IQR, 19.6–75 years) and 120 male participants with a median age of 58 years (IQR, 30.6–75 years). The median weight for females was 60.5 kg (IQR, 51–71 kg) and 76 kg (IQR, 68–86 kg) for females. The median height for females was 162 cm (IQR, 152–170 cm) and 174 cm (IQR, 168–179 cm) for males. Table 1 gives the distribution of CV risk factors.

### 3.2. Arterial Elongation

The median length of the infrarenal aorta was 92.4 mm (IQR, 83.4–100.1 mm), the median length of the left CIA was 63.7 mm (IQR, 48.3–74.5 mm), the median length of the left EIA was 107 mm (IQR, 94.9–122 mm), the median length of the right CIA was 63.1 mm (IQR, 50.5–74.4 mm), and the median length of the right EIA was 108.3 mm (IQR, 94.4–120.6 mm). Descriptive statistics are presented in Table 2, while linear regression and correlation results are outlined in Table 3 and Table 4.

Male CIAs and EIAs showed higher TI values on both sides than females (left CIA, t(202) = −2.572, *p* = 0.011; left EIA, t(202) = −3.615, *p* < 0.001; right CIA, t(202) = −2.284, *p* = 0.023; and right EIA, U = 3579, *p* < 0.001). A significant positive correlation was observed between the TI value of all arterial segments and age (infrarenal aorta, F [1, 202] = 221.757, *p* < 0.001; left CIA, F [1, 202] = 170.517, *p* < 0.001; left EIA, F [1, 202] = 200.533, *p* < 0.001; right CIA, F [1, 202] = 181.248, *p* < 0.001; and right EIA, F [1, 202] = 189.674, *p* < 0.001) (Table 3). The TI of the infrarenal aorta was significantly associated with CKD (*r* = 0.157, *p* = 0.027). A weak negative correlation was found between right EIA tortuosity and obesity (*r* = −0.145, *p* = 0.041) (Table 4).

The EIA of males had higher AAC values than females on both sides (left, U = 3636, *p* < 0.001; right, U = 3689, *p* = 0.001). A significant positive correlation was noted between the AAC value of all arterial segments and age (infrarenal aorta, F [1, 202] = 97.094, *p* < 0.001; left CIA, F [1, 202] = 102.157, *p* < 0.001; left EIA, F [1, 202] = 128.158, *p* < 0.001; right CIA, F [1, 202] = 88.413, *p* < 0.001; and right EIA, F [1, 202] = 132.165, *p* < 0.001) (Table 3). The AAC of the infrarenal aorta was significantly associated with CKD (*r* = 0.173, *p* = 0.016), while the AAC of both CIAs was significantly associated with hypertension (left, *r* = 0.157, *p* = 0.027; right, *r* = 0.179, *p* = 0.012). A weak negative correlation was revealed between the AAC of the right CIA and hyperlipidemia (*r* = −0.149, *p* = 0.037), between the AAC of the left EIA and smoking (*r* = −0.162, *p* = 0.020), and between the AAC of the right EIA and obesity (*r* = −0.170, *p* = 0.019) (Table 4).

### 3.3. Geometry of Aortic Bifurcation

Descriptive statistics are presented in Table 5, while linear regression and correlation results are outlined in Table 6 and Table 7. Males showed higher radius discrepancy values than females (U = 3722, *p* = 0.001). A significant positive correlation was observed between aortic bifurcation angle and age (F [1, 202] = 12.525, *p* < 0.001), between both CIA planarities and age (left, F [1, 202] = 14.919, *p* < 0.001; right, F [1, 202] = 9.664, *p* = 0.002), between aortic bifurcation asymmetry and age (F [1, 202] = 10.744, *p* = 0.001), and between radius discrepancy and age (F [1, 202] = 37.024, *p* < 0.001). (Radius discrepancy: the extent of deviation from the optimal bifurcation [Table 6].) The take-off angle of the right CIA was significantly related to CKD (*r* = 0.153, *p* = 0.031), while the planarity of the left CIA was significantly related to hyperlipidemia (*r* = 0.195, *p* = 0.006) (Table 7).

## 4. Discussion

To the best of our knowledge, we are the first to study the effect of sex on AI segment elongation; however, sex-related arterial changes have already been assessed in other regions. For example, Tawfik et al. [18] found no relationship between the TI of the thoracic aorta determined on CTA images and sex in 182 individuals. In contrast, in a study involving 345 patients, Togay-Işikay et al. [19] observed 85 elongation-related carotid abnormalities (kinking and coiling) on duplex ultrasonography with a higher prevalence in females (*n* = 60) than in males (*n* = 25). Similarly, in a cohort of 870 patients undergoing invasive coronary angiography, Chiha et al. [20] showed that females were more likely to have severe coronary tortuosity than males. On the other hand, Wood et al. [21] noted a clear male predominance in the elongation of the superficial femoral arteries on MRA images in a sample of 18 young volunteers. We also revealed that AI segment tortuosity is more common in males than in females. Sex seems to have different consequences for arterial sites. Further research is needed to define the exact mechanism by which sex influences the structural integrity of the arterial wall.

We found that age is a significant factor in the elongation of the AI segment. So far, only one study has investigated the impact of age on the tortuosity of the AI segment. Song et al. [22] examined the TI of CIAs and EIAs on CTA images in individuals with (*n* = 110) and without (*n* = 59) abdominal aortic aneurysm and showed that the TI of the iliac arteries was positively associated with age in both groups. Age-related arterial elongation has also been analyzed in other locations such as the thoracic aorta, carotid arteries, and coronary arteries. Belvroy et al. [23] observed a significant difference in the TI of the thoracic aorta on CTA images between two groups: one containing 100 subjects under 65 years of age and the other containing 100 subjects over 65 years of age. The TI was higher in the latter group. Similarly, in a study by Tawfik et al. [18], the authors noted a moderate positive correlation between the TI of the thoracic aorta and age. Thomas et al. [24] compared the geometric characteristics of the carotid arteries of 25 younger individuals (19–38 years old) with those of 25 older individuals (63 ± 10 years old). The comparison was performed using a peripherally gated black-blood MRI protocol, which revealed higher TI values in older individuals. Sun et al. [25] also investigated the TI of carotid arteries on the time-of-flight MRA images of 247 individuals without neurological disease. The patients were divided into two groups—a young group (≤60 years) and an older group (>60 years)—and significantly higher TI values were reported in the older group. Khosravani-Rudpishi et al. [26] evaluated coronary angiograms of a cohort of 737 patients and described a positive association between coronary tortuosity and age. Roach et al. [27] hypothesized that with age, the amount of collagen fibers increases while their unstretched length decreases in the arterial wall, allowing cross-links and adhesions to form, which can then lead to arterial elongation.

In our study, there was a negative relationship between the elongation parameters of the right EIA and obesity (BMI > 30 kg/m^2^). Li et al. [28] looked at the effect of obesity (BMI > 28 kg/m^2^) on the tortuosity of coronary arteries detected on CTA images of 1280 participants and showed that the higher the BMI, the less elongated the coronary arteries. On the other hand, Wang et al. [29] mentioned a significant positive correlation between the tortuosity of the extracranial segment of the internal carotid arteries on digital subtraction angiography images and BMI in a group of 116 patients.

Contrary to our results that found a negative association between the AAC of the left EIA and smoking, neither Wang et al. [29] nor Lauric et al. [30] observed such a correlation; however, they examined the internal carotid arteries rather than the iliac arteries. Lauric et al. [30] measured the curvature of the carotid arteries in 130 patients and derived it from 3D rotational-angiography volumes. One explanation for smoking inhibiting arterial elongation may be that smoking reduces the amount of both type I collagen and decorin in the arterial wall, as described by Faarvang et al. [31].

Only a limited number of publications provide comprehensive information on the role of hypertension in arterial elongation. Li et al. [32] reported increased coronary tortuosity in conjunction with essential hypertension in a group of 1010 patients. They performed CTAs to visualize the tortuosity of the coronary arteries. The researchers also used biomechanical models to demonstrate that arteries can become elongated due to high internal pressure [33]. These observations are consistent with our findings on the link between CIA elongation and hypertension. Arribas et al. [34] suggest that high blood pressure causes fatigue and an accelerated degradation of elastic fibers, leading to the loss of resilience of the arterial wall and a longitudinal elongation of the vessel.

In our study, there was a negative relationship between right CIA elongation and hyperlipidemia. In the case of coronary arteries, Li et al. [32] published a negative association, while Groves et al. [35] did not notice any correlation between tortuosity and hyperlipidemia. Groves et al. [35] included 1221 patients who had invasive coronary angiography in their study. The mechanism of hyperlipidemia in arterial elongation is unclear, but nevertheless, these data suggest that hyperlipidemia does not promote arterial elongation.

We are the first to identify a significant positive association between infrarenal aortic elongation and CKD. Chronic kidney disease is known to be involved in the degradation of elastin in the arterial wall and, as a result, in its stiffening [36]. The degradation of elastin can trigger the mechanical instability and tortuosity of the arterial wall [37].

Lakchayapakorn et al. [38] evaluated 65 cadavers and found the mean angle of the aortic bifurcation to be 54°. Our study had similar results, with a mean value of 53°. Sun et al. [39] assessed the planarity of the aortic bifurcation in 20 healthy individuals. According to their results, the planarity angles of the aortic bifurcation varied between 0.3° and 24.4°. These findings are in agreement with our results, which showed a range of 0.08° to 31.93° for the left CIA and 0.13° to 29.05° for the right CIA.

Shakeri et al. [14] investigated the impact of age on the angle of the aortic bifurcation in a group of 59 patients undergoing digital subtraction angiography. In contrast to our observations, they did not reveal a connection between bifurcation angle or bifurcation asymmetry and age. Thomas et al. [24]; however, noted a significant effect of age on the carotid bifurcation angle, as did Jeon et al. [40], who analyzed this from MRA images of 300 patients.

In our study, a significant positive correlation between left CIA planarity and hyperlipidemia was confirmed. Apart from our study, the effect of hyperlipidemia on the angles of the aortic bifurcation has not been addressed.

We detected a significant positive association between the right CIA take-off angle and CKD. We hypothesize that the relationship between the right CIA take-off angle and CKD is due to changes induced by the elongation of the infrarenal aorta. This hypothesis is supported by the fact that the statistical significance of this correlation decreases when the effect of TI or AAC on the infrarenal aorta is eliminated. No one else has studied the implications of CKD in this context.

Our results indicate that sex and age contribute to the deviation from the optimal aortic bifurcation geometry described by Murray’s law. We assume that deviation from optimal bifurcation disrupts blood flow and potentially creates the conditions for plaque formation. This hypothesis is corroborated by a publication of Schoenenberger et al. [41], who examined the deviation from Murray’s law in coronary bifurcations in a group of 253 patients. They showed that the extent of deviation from Murray’s law corresponded with densely calcified plaques proximal and distal to the coronary bifurcations.

In conclusion, sex, age, and certain CV risk factors have a significant impact on the geometry of the AI segment. However, further studies are needed to assess the clinical relevance of the geometric differences. We plan to investigate the role of geometric parameters in the development of aortoiliac stenosis/occlusion or dilatation. We also intend to analyze the extent to which geometric variables determine the planning, device selection, feasibility, and efficacy of aortoiliac interventions performed through the common femoral artery.

## Figures and Tables

**Table 1:** Distribution of cardiovascular risk factors.

Cardiovascular Risk Factors/ Comorbidities, *n* (%)	All Participants (*n* = 204)	Female Participants (*n* = 84)	Male Participants (*n* = 120)
Obesity	32 (15.7)	10 (11.9)	22 (18.3)
Smoking	25 (12.3)	9 (10.7)	16 (13.3)
Hypertension	104 (51.0)	35 (41.7)	69 (57.5)
Diabetes mellitus	40 (19.6)	14 (16.7)	26 (21.7)
Hyperlipidemia	71 (34.8)	25 (29.8)	46 (38.3)
CKD	14 (6.9)	7 (8.3)	7 (5.8)

CKD—chronic kidney disease.

**Table 2:** Descriptive statistics of elongation parameters.

Elongation Parameters	All Participants (*n* = 204)	Female Participants (*n* = 84)	Male Participants (*n* = 120)
Tortuosity index (%), median (IQR)			
Aorta	0.862 (0.403–2.187)	0.785 (0.323–1.757)	0.967 (0.427–2.500)
Left CIA	1.731 (0.753–4.306)	1.191 (0.635–3.081)	2.178 (0.918–6.295)
Left EIA	2.856 (0.946–8.020)	2.049 (0.680–4.567)	4.132 (1.325–11.058)
Right CIA	0.823 (0.257–2.611)	0.478 (0.198–2.123)	0.956 (0.387–3.803)
Right EIA	3.048 (1.289–12.139)	2.122 (0.929–6.180)	3.970 (1.664–16.335)
Average absolute curvature (×1000), median (IQR)			
Aorta	6.665 (4.740–10.067)	6.305 (4.865–9.383)	7.095 (4.672–10.238)
Left CIA	10.275 (6.395–16.207)	9.660 (5.610–15.470)	10.700 (6.888–17.040)
Left EIA	12.640 (7.510–22.225)	10.375 (6.872–17.948)	16.190 (8.477–25.733)
Right CIA	11.555 (8.638–18.580)	10.520 (8.595–16.082)	12.860 (8.775–19.712)
Right EIA	11.900 (7.672–24.992)	9.830 (6.750–19.267)	15.420 (8.430–26.843)

CIA—common iliac artery; EIA—external iliac artery; IQR—interquartile range.

**Table 3:** Correlation between elongation parameters and age.

Elongation Parameters	*r*-Value	Beta ± SD	*p*-Value
Tortuosity index			
Aorta	0.723	0.031 ± 0.002	<0.001
Left CIA	0.664	0.032 ± 0.003	<0.001
Left EIA	0.700	0.035 ± 0.003	<0.001
Right CIA	0.688	0.040 ± 0.003	<0.001
Right EIA	0.696	0.034 ± 0.002	<0.001
Average absolute curvature			
Aorta	0.570	0.010 ± 0.001	<0.001
Left CIA	0.580	0.013 ± 0.001	<0.001
Left EIA	0.623	0.014 ± 0.001	<0.001
Right CIA	0.552	0.010 ± 0.001	<0.001
Right EIA	0.629	0.015 ± 0.001	<0.001

CIA—common iliac artery; EIA—external iliac artery; SD—standard deviation.

**Table 4:** Correlation between elongation parameters and cardiovascular risk factors.

Elongation Parameters, *r*-Value	Obesity	Smoking	Hypertension	Diabetes Mellitus	Hyperlipidemia	CKD
Tortuosity index						
Aorta	−0.039	0.031	0.006	−0.092	−0.008	0.157 *
Left CIA	0.051	0.032	0.070	0.038	−0.136	−0.005
Left EIA	−0.077	−0.123	0.058	0.080	−0.051	0.067
Right CIA	0.089	0.021	0.120	−0.036	−0.113	0.081
Right EIA	−0.145 *	−0.065	0.074	0.090	−0.071	0.021
Average absolute curvature						
Aorta	−0.091	−0.006	0.030	−0.034	0.074	0.173 *
Left CIA	0.058	−0.057	0.157 *	−0.099	−0.104	0.075
Left EIA	−0.125	−0.162 *	0.147	0.075	−0.070	0.033
Right CIA	−0.036	−0.064	0.179 *	0.031	−0.149 *	0.049
Right EIA	−0.170 *	−0.112	0.122	0.111	−0.130	0.048

CIA—common iliac artery; CKD—chronic kidney disease; EIA—external iliac artery. * represents *p* < 0.05.

**Table 5:** Descriptive statistics of aortic bifurcation metrics.

Aortic Bifurcation Metrics	All Participants (*n* = 204)	Female Participants (*n* = 84)	Male Participants (*n* = 120)
Angles (°), median (IQR)			
Left CIA take-off angle	25.39 (20.48–32.03)	25.27 (19.01–31.32)	25.39 (21.43–32.22)
Right CIA take-off angle	26.20 (21.50–31.71)	25.77 (20.96–30.09)	26.26 (22.13–32.45)
Aortic bifurcation angle	52.32 (46.06–59.30)	51.20 (45.04–58.75)	52.49 (47.90–60.56)
Left CIA planarity	5.91 (2.54–10.27)	6.15 (2.54–9.25)	5.79 (2.53–10.79)
Right CIA planarity	5.94 (2.34–9.49)	4.97 (2.12–9.33)	6.48 (2.63–9.54)
Aortic bifurcation angle asymmetry	0.01 (−0.16–0.19)	0.01 (−0.16–0.19)	0.01 (−0.16–0.19)
Cross-sectional area (cm^2^), median (IQR)			
Distal infrarenal aorta	159.46 (108.42–194.92)	115.90 (89.28–145.55)	189.95 (143.85–223.37)
Proximal left CIA	69.05 (40.56–88.68)	49.90 (32.85–59.00)	82.46 (52.45–103.65)
Proximal right CIA	74.66 (39.78–88.61)	50.66 (32.79–64.70)	91.46 (51.52–100.01)
Radius discrepancy (cm), median (IQR)	1.12 (0.73–1.48)	0.99 (0.63–1.32)	1.26 (0.91–1.60)

CIA—common iliac artery; IQR—interquartile range.

**Table 6:** Correlation between aortic bifurcation metrics and age.

Aortic Bifurcation Metrics	*r*-Value	Beta ± SD	*p*-Value
Angles			
Left CIA take-off angle	0.064	0.001 ± 0.001	0.365
Right CIA take-off angle	0.093	0.001 ± 0.001	0.186
Aortic bifurcation angle	0.242	0.002 ± 0.001	<0.001
Left CIA planarity	0.262	0.009 ± 0.002	<0.001
Right CIA planarity	0.214	0.008 ± 0.002	0.002
Aortic bifurcation angle asymmetry	0.225	0.001 ± 0.001	0.001
Radius discrepancy	0.394	0.009 ± 0.002	<0.001

CIA—common iliac artery; SD—standard deviation.

**Table 7:** Correlation between aortic bifurcation metrics and cardiovascular risk factors.

Aortic Bifurcation Metrics, *r*-Value	Obesity	Smoking	Hypertension	Diabetes Mellitus	Hyperlipidemia	CKD
Angles						
Left CIA take-off angle	0.010	−0.029	−0.019	−0.046	0.119	−0.111
Right CIA take-off angle	−0.039	0.047	0.054	−0.019	−0.094	0.153 *
Aortic bifurcation angle	−0.019	0.024	0.001	−0.063	0.027	0.041
Left CIA planarity	0.078	−0.010	−0.030	0.061	0.195 **	0.041
Right CIA planarity	0.052	−0.045	−0.086	−0.018	0.095	0.098
Aortic bifurcation angle asymmetry	0.055	−0.014	−0.107	0.056	0.119	0.062
Radius discrepancy	−0.016	0.116	0.026	0.002	0.052	−0.080

CIA—common iliac artery; CKD—chronic kidney disease. *, *p* < 0.05; **, *p* < 0.01.

## Data Availability

The data presented in this study are available upon request from the corresponding author. The data are not publicly available due to reasons pertaining to patient privacy.
